# Vocabulary size and structure affects real-time lexical recognition in 18-month-olds

**DOI:** 10.1371/journal.pone.0219290

**Published:** 2019-07-11

**Authors:** Arielle Borovsky, Ryan E. Peters

**Affiliations:** Speech, Language and Hearing Sciences, Purdue University, West Lafayette, Indiana, United States of America; Tohoku University, JAPAN

## Abstract

The mature lexicon encodes semantic relations between words, and these connections can alternately facilitate and interfere with language processing. We explore the emergence of these processing dynamics in 18-month-olds (N = 79) using a novel approach that calculates individualized semantic structure at multiple granularities in participants’ productive vocabularies. Participants completed two interleaved eye-tracked word recognition tasks involving semantically unrelated and related picture contexts, which sought to measure the impact of lexical facilitation and interference on processing, respectively. Semantic structure and vocabulary size differentially impacted processing in each task. Category level structure facilitated word recognition in 18-month-olds with smaller productive vocabularies, while overall lexical connectivity interfered with word recognition for toddlers with relatively larger vocabularies. The results suggest that, while semantic structure at multiple granularities is measurable even in small lexicons, mechanisms of semantic interference and facilitation are driven by the development of structure at different granularities. We consider these findings in light of accounts of adult word recognition that posits that different levels of structure index strong and weak activation from nearby and distant semantic neighbors. We also consider further directions for developmental change in these patterns.

## Introduction

The mature lexicon encodes relations between words along numerous dimensions, including semantic similarity. Lexico-semantic structure, in turn, interacts with language processing. For instance, semantically related concepts can alternatively facilitate or interfere with language processing in adults[1]. Typically, our theoretical understanding of lexico-semantic dynamics has been informed from findings in the adult psycholinguistic literature, which has explored lexical processing in individuals who know many thousands of words and have correspondingly complex semantic networks. Less is known regarding how structure initially emerges and interacts in language processing, although understanding this process has the potential to provide crucial insight into how learners of all ages represent and interpret connections among word meanings. Fundamental questions include: How does lexico-semantic structure initially develop? When does nascent structure in the emerging lexicon interact with real-time linguistic processing?

We focus here on the interplay of lexico-semantic structure and language processing in semantically related and unrelated contexts in 18-month-old toddlers. The interaction of lexical structure and processing is particularly interesting in 18-month-olds, who show significant variability in vocabulary composition and size, and are entering a period of rapid vocabulary growth [2,3]. Importantly, semantic priming studies of 18- to 24-month-olds point to a developmental shift in semantic structure of the lexicon in the latter half of the second year of life. While 24-month-olds show robust semantic priming in a variety of paradigms [4,5], 18- to 21-month-olds do not[6–8], suggesting that children are actively developing semantic connections in their lexicon at these ages. Further, inconsistent priming patterns in 18- to 20-month-olds seem to be connected to vocabulary size. Children in this 18- to 20-month age range with relatively larger vocabularies tend to show robust semantic priming effects while those with smaller vocabularies do not [7,8]. Together, these findings suggest that semantic priming is driven by several factors including maturational development and growth of the child’s lexicon. A larger lexicon, simply by chance, is likely to include semantically overlapping concepts, whereas a child with a smaller lexicon will have fewer semantic connections among word meanings.

Thus, there is compelling evidence that emergent semantic structure begins to modulate lexical activation somewhere around the middle of the second year of life, though much is still unknown regarding the mechanisms that support these processes. Building a comprehensive account of early lexical processing and representation has the potential to constrain theories of the developmental dynamics of lexical activation and structure, and can provide practical insight into early word learning processes. We therefore explore these questions in a large sample (N = 79) of 18- to 20-month-old children, by measuring lexical recognition in an eye-tracked looking-while-listening task with semantically related and unrelated picture pairs.

Eye-tracking studies of lexical recognition in two-year-olds support the notion that vocabulary size and structure shape lexical recognition from an early age. Twenty-five month-olds with larger productive vocabularies are faster to interpret spoken words in this paradigm than children with smaller vocabularies[9]. Similarly, vocabulary structure in 24-month-olds influences real-time recognition of known and novel words [10,11], such that words in semantically denser categories are learned and recognized more efficiently than words in sparser categories. Together, these findings suggest that building lexico-semantic networks may support language processing and vocabulary growth, though this has yet to be verified in younger populations.

A primary limitation of prior studies linking early lexico-semantic structure to word learning and processing is that they have relied on relatively coarse metrics of semantic density or semantic relations at a categorical level. The adult psycholinguistic literature, however, suggests that processing can be influenced by semantic structure at different levels of granularity. For example, directly related concepts (that share association or feature overlap) prime each other–such as concepts like *cat* and *dog*, but priming can also be observed between concepts that do not share associative or featural connections, as in indirect priming (e.g. *lion* primes *stripes* via an intermediate connection *tiger*)[12,13]. Similarly, adults in a variety of word-recognition tasks (e.g. lexical decision and categorization) experience interference (i.e. slower RTs) when words have many near neighbors, while words with many distant neighbors experience lexical facilitation (i.e. faster RTs)[14]. Thus, adult lexical processing is influenced by semantic neighborhood structure at various levels.

Therefore, the current study addresses whether and how semantic structure at different levels associates with lexical processing in young children. Our approach has a number of necessary differences from prior adult studies, largely because 18-month-olds’ vocabularies are limited compared to adults. Though this limited lexical knowledge precludes implementation of adult-like metrics to study the effects of semantic structure on lexical processing, it also imparts unique benefits. Unlike adults, it is possible to capture a sizeable snapshot of toddlers’ productive vocabulary, thereby enabling the measurement of semantic structure at the *individual child level*–rather than estimating structure over the population as is typically done in adults. To this end, we employ semantic graph network analyses to measure semantic connectivity over each child’s total productive lexicon and at the word-level[15–17].

Graph-theoretic modeling techniques have already yielded exciting insights into the structure of toddler lexicons[16,18–20]. For example, longitudinal modeling with graph network metrics has highlighted that early productive vocabularies are not randomly acquired, but grow in semantically structured pattern[16]. Further, semantic structure may support overall productive vocabulary growth: children with early delays in productive vocabulary acquisition, such as late-talking children, have less semantically-structured lexicons than children without delays [18]. However, there has not been direct empirical evidence that these metrics predict toddlers’ linguistic processing. Establishing whether such a link exists is the primary goal of the current study. We therefore carry out an analysis that explores, for the first time, how several indices of semantic structure at the word-level, category-level and lexicon-level influence real-time recognition and activation of word meanings in 18- to 20-month-old toddlers.

Below, we outline our approach to measuring vocabulary size and structure, before enumerating the potential outcomes and theoretical implications of this investigation.

### Productive vocabulary size

Productive vocabulary is measured using the MacArthur Bates Communicative Developmental Inventory, Words and Sentences (MBCDI)[21]. This instrument includes a parental checklist of words that are commonly spoken by young children between the ages of 16 to 30 months, and, at 18 months, captures a relatively comprehensive snapshot of the child’s overall lexicon [22]. Moreover, this measure of productive vocabulary has been reliably associated with numerous group and individual differences in lexical processing in 18-month-old children[7,9]. Because productive vocabulary size associates strongly with the child’s overall lexicon and other aspects of word recognition, we use the term “vocabulary size,” “lexicon” and “productive vocabulary” interchangeably throughout the paper.

### Vocabulary structure

In this study, we measure, for the first time, how individual productive vocabulary structure influences real-time word recognition in 18-month-old toddlers. Vocabulary structure is measured at three levels: category-level, word-level and lexicon-level structure.

The *category-level* measure is calculated using a procedure adopted in two recent studies of semantic density in 24-month-olds[10,11]. This category-level semantic density metric identifies higher and lower semantic density domains for each child by measuring the proportion of total words that child produces on the MBCDI for each of six early-acquired semantic categories. By identifying individual children’s highest and lowest density categories, this measure captures relative differences in semantic density while controlling for overall productive vocabulary size, and represents a medium grain-sized measure of semantic density.

The *word-level* and *lexicon-level* semantic structure measures are derived using graph theoretic techniques that enable the measurement of the impact of semantic structure on semantic processing at a finer and broader scale than has been previously possible using category-level density measures. While these techniques for measuring semantic structure are not new [17], and have been applied to the study of other aspects of toddler word-learning[19,20,23], no investigation to date has explored whether and how these graph-theoretic derived measures contribute to real-time lexical processing. We identify two measures that reflect word-level and lexicon-level structure–degree centrality and global clustering coefficient, respectively. These measures are described below.

The word-level measure of semantic structure, degree, simply reflects the number of directly connected semantic neighbors for each lexical item. In graph theory, this measure has been used as a metric of the relative importance of nodes in their potential communication activity [24]. In the current context of lexico-semantic networks, “communication” reflects a potential for spreading activation from nearby neighbors, and thus degree is relevant to language processing in semantically related and unrelated contexts.

Lexicon-level semantic structure is measured by deriving a graph-theoretic metric of the overall semantic connectivity of a child’s lexicon, global clustering coefficient. Global clustering coefficient characterizes the average level of connectivity in the neighborhoods of words across the network, and represents the probability that two neighbors of a randomly chosen word will be connected–reflecting the potential connectivity of near and far lexical neighbors. High interconnectivity in neighborhoods, and thus high global clustering coefficient, is argued to play an important functional role in enabling fluent word processing and retrieval in mature lexico-semantic networks [17,25].

Previous studies using graph-theoretic modeling in early vocabulary development have formalized semantic connections between words using a variety of measures including free association norms [16]; co-occurrence statistics from corpora of child-directed speech [18] and semantic feature norms [16,23]. While each of these methods have their own strengths and weaknesses, we take the latter feature norm approach with some important modifications. Prior work with feature norms has suffered from the lack of available feature production norms that cover all noun items in the MBCDI. In fact, in prior work, norms were only available for 130 out of 359 nouns, and these items were not equally distributed across categories, therefore over-representing some categories, like animals (72.1% available), and under-representing others, such as people (missing entirely). We use norms that result from a recent effort to develop feature norms for every noun that appears on the MBCDI [26] [27]. Feature norms sets have been developed by asking adults to provide features that come to mind for a number of English nouns. The features provided for each concept were then further tagged with numerous semantic dimensions, and classified as one of four broad feature types: perceptual, functional, taxonomic and encyclopedic features[28]. Prior work with a limited set of normed MBCDI items had measured semantic network metrics using only perceptual and functional features, which were likely to be available to a child’s direct experience [16,23], and we follow this approach in the current paper.

### Lexical recognition

We use an adaptation of the looking-while-listening (LWL) task to measure the impact of semantic structure on real-time lexical recognition. This task uses children’s gaze toward named visual referents on a screen as an index of real-time interpretation of word meanings and has been employed with children as young as 6–9 months of age [29]. Performance in this paradigm is often characterized to reflect linguistic “processing speed”[30] which incorporates a variety of cognitive processes, such as auditory processing and lexical activation. Importantly, gaze-derived measures of toddlers’ word recognition is sensitive to differences in productive vocabulary size[9,31] and structure [10,11].

Though prior work highlighting individual differences in lexical processing using the LWL task has often presented pairs of unrelated visual referents, performance when the target and distractors are related can illuminate important processes in lexical activation. Phonological overlap between object pairs can delay target recognition when objects share a phonetic onset (DOG-DOLL), but not phonetic rhyme (BALL-DOLL), suggesting that young children activate and disambiguate words incrementally[32]. More recently, numerous eye-tracking studies with adults and children have adopted a similar chain of logic to demonstrate a multitude of ways in which word meanings are encoded and activated, including among phonological, categorical, associative, thematic, and perceptual dimensions[33].

### The current study

We measured the impact of semantic structure on lexical recognition in two experimental tasks comprising semantically unrelated and semantically related picture pairs. Prior research suggests that denser semantic structure (at the category-level) in 24-month-olds can facilitate word recognition in unrelated picture contexts[10]. Correspondingly, we expected to find a facilitatory effect of category-level density on word recognition in unrelated trials. Additionally, we planned to explore how different levels of structure might influence processing in unrelated trials, though, given that no prior studies in children had addressed this particular question, we did not have a priori expectations regarding how structure would interact with vocabulary in this condition.

In semantically related contexts, we expected that the presence of a semantic competitor should interfere with target recognition, as in prior word recognition studies in adults [34,35]. Specifically, we expect this pattern to reflect several (non-mutually exclusive) processes in lexical recognition, characterized by Yee and Sedivy (2006)[35]. First, the presence of two semantically related competitors should lead to co-activation of conceptual overlap among the two images, this conceptual co-activation should increase the time it takes to select the labeled object, and inhibit the unlabeled object. For brevity throughout, we will refer to these conjoint processes in lexical recognition (co-activation and inhibition) during semantically related trials as “interference” through the paper. We additionally expected this interference pattern to associate with productive vocabulary size, following prior priming studies that have demonstrated an absence of semantic priming effects in 18-month-olds with relatively smaller vocabularies[7]. For children with larger vocabularies, density at each of the levels of semantic structure is likely to influence processing differentially. One possibility, suggested by adult processing, is that local connections between words (at the word and category level) associate with lexical interference. This outcome would indicate that 18-month-olds with relatively larger vocabularies are rapidly recognizing and activating semantic links among word meanings that share many related features. Alternatively, as even the most lexically proficient 18-month-olds still have relatively small lexicons when compared to adults, they may still be developing appropriate semantic boundaries between word meanings at multiple lexical levels[36]. In this case, we might expect that higher-level metrics of vocabulary size that include more distant semantic connections (i.e., category- and lexicon-level metrics) should associate with interference effects. Therefore, the inclusion of related and unrelated experimental conditions will yield important insights into how mechanisms of semantic facilitation and interference emerge during a pivotal period of lexical development.

## Methods

### Participants

18- to 20-month-olds were recruited over a two-year window as part of a larger project to explore how lexico-semantic structure predicts later language outcomes. Of this sample, 101 participated in the eye-tracking task and their parent completed an MBCDI. Seventy-eight children were retained in the final analyses (35 F, 44 M, age range: 18.02–21.38 months, median age: 18.57 months). The remaining 23 children were not included for the following reasons: falling >1.5 SD below the mean on the cognitive subtest of the Bayley Scales of Infant Toddler Development (N = 1), failure to meet language criteria (N = 3), parent reported suspected uncorrected hearing or vision concern (N = 4), or child was receiving services for a motor, speech or language concern (N = 5). Additional children were then removed if they experienced fussiness, data loss or equipment errors that resulted in < 2 trials in each experimental condition (N = 10), All remaining children were reported to have no hearing or vision concerns, and primarily learning English (hearing no more than 20 hours a week of a language other than English). Mother’s education was distributed as follows: high school or less (N = 5), some college (N = 10), college degree (N = 30) advanced degree (N = 32), not reported (N = 2). Most parents also reported information on race and ethnicity (N = 68), with 68.4% (N = 54) identifying as White, 11.4% (N = 9) as African American, 6.2% (N = 5) as multi-racial or other. Further, 10.1% (N = 8) identified as Hispanic/Latino. Parents or guardians of children in the study provided written informed consent for participation and the Florida State University IRB approved the study (#2017.21274).

### Materials

#### Category and item selection

The vocabulary items on the MBCDI: Words and Sentences, include a number of early-acquired categories which have been used in prior semantic priming and lexical processing studies [7,10,11]. Following prior work using category density metrics in lexical recognition, we selected six category domains with items that appear in 18-month-old productive vocabularies, as verified from the Cross-Linguistic Lexical Norms database (CLEX)[37]. The category domains selected for the experimental task were: ANIMALS, CLOTHING, VEHICLES, BODY-PARTS, along with two food sub-categories: FRUIT and DRINKS. The number of items that appear on the CDI checklist in each of these subcategories respectively is: 43, 28, 14, 27, 7, and 7.

We selected two words from each category domain to be included in the experimental materials. Selected items were produced by at least 70% of 18-month-old children. These items were: ANIMALS: Dog, Bird; FRUIT: Banana, Apple; CLOTHING: Shoe, Diaper; VEHICLES: Car, Airplane; DRINKS: Juice, Milk; BODY-PARTS: Nose, Mouth. In related trials, each image was paired with the other item from the same category domain. In unrelated trials, each image was paired with another item from another category. These pairings were: Banana-Juice, Apple-Milk (FRUIT-DRINK); Car-Shoe, Airplane-Diaper (VEHICLES-CLOTHING); Nose-Bird, Teeth-Dog (BODY PARTS-ANIMALS). Each participant saw all related and unrelated picture pairings in the experimental task.

Our rationale in selecting the particular categories and category pairing were three-fold. First, as a major goal of this study is to explore how both previously used category density metrics and graph-theoretic metrics of semantic structure contribute to word recognition in toddlers, we sought to select items from categories that had been used in prior studies using the category density measure[10]. Secondly, our experimental design necessitated the selection of items from general nominal categories which are commonly known by 18-mo-old toddlers, and for which we could find relatively prototypical images that could be recognized by toddlers of this age. Thirdly, for the unrelated task, we sought to pair items from categories that were matched for relative salience and interest. Through earlier piloting work and consultation with lab members, we determined that animal and vehicles elicited strongest visual interest in toddlers, while body-parts and clothing generated the least (though all items were age-appropriate and interesting).

#### Auditory stimuli

We recorded spoken stimuli for all experimental items in infant-directed speech from a female Standard American English speaker, on a mono channel at 44.1 kHz sampling rate. Durations of all lexical stimuli were adjusted to a mean length of 1020 ms. In addition to the experimental items, the speaker also recorded a number of encouraging phrases to help maintain toddlers’ interest in the experiment. These included tag phrases that appeared after the lexical items were completely spoken within each experimental trial such as, “Do you like it?” and, “That’s cool!” Other pre-recorded encouraging phrases were included in break trials like, “These pictures are fun!” and, “You’re doing great!” Additionally, each experimental trial began with a recording of the speaker saying “Look” at the onset of the gaze-contingent center stimulus before the onset of the spoken word. All lexical items, tag phrases, encouraging phrases, and attention getters were normalized to a standard mean intensity of 70 dB to ensure that all auditory stimuli were presented at stable levels across the study.

#### Visual stimuli

**Fig 1:**
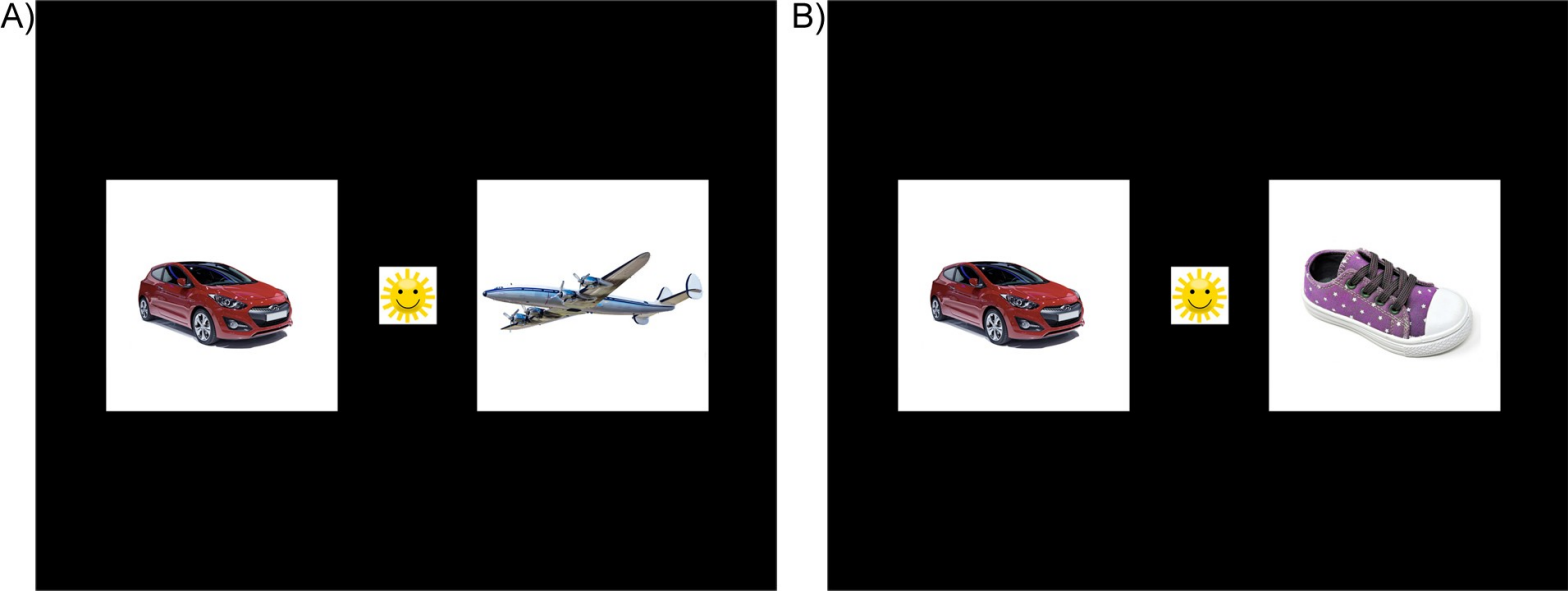
Illustration of visual stimuli in experimental trials. In (A) Semantically related trials, where Target and Distractor images were semantically related, and (B) Semantically unrelated trials, where Target and Distractor did not share category membership. Each trial began with a preview period, where the Target and Distractor images appeared alone on the left and right side of the monitor. Next, a small, colorful center stimulus (e.g., smiling sunshine) appeared, with an auditory stimulus (*Look*!). Once the infant fixated on the center stimulus for 100 ms, the center image automatically disappeared, and the label for the target image was spoken, (e.g., *Shoe*! *That’s cool*!*)* Due to licensing restrictions, similar, but not identical images of depicted objects appeared in the study. Car image courtesy of User:Thesupermat / Wikimedia Commons / CC-BY-SA-3.0 (background removed). Remaining illustrated images courtesy of https://www.maxpixel.net//CC01.0.

Experimental items appeared as photorealistic color images of 400 x 400 pixels in size (on a 1280 x 1024-pixel screen). When possible, images were isolated on white backgrounds, but in some cases, such as with body-parts like ‘nose’ it was not feasible to present the item in isolation from the body while maintaining item recognizability. Images were chosen to be representative of pictures seen from a young child’s point of view, as verified by consulting with other parents and laboratory members. Fig 1 provides a sample illustration of visual stimuli in related and unrelated trials. Additional images were also selected to maintain the child’s interest. These included large images such as a full-screen image of the sesame street character, Elmo, and other small images such as smiling faces to direct fixations towards the screen.

### Procedure

#### Offline assessments

**Fig 2:**
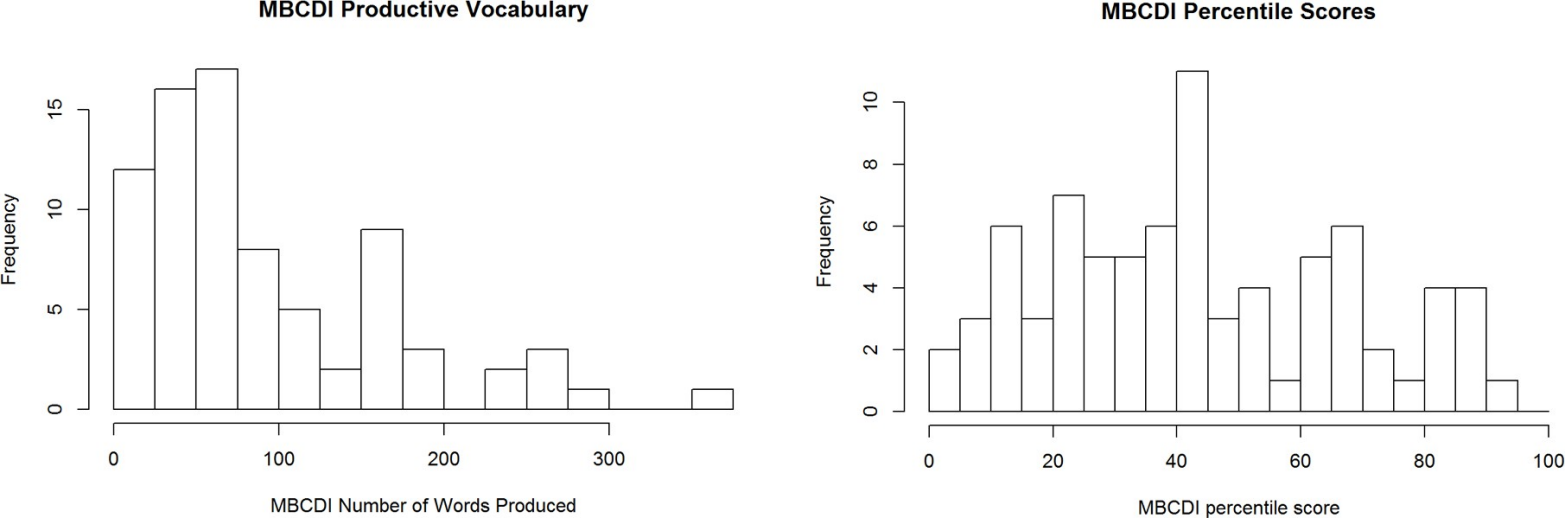
Distribution of raw productive vocabulary scores and percentile vocabulary scores on MBCDI for all participants.

**Background and vocabulary measurement.** Before beginning the experiment, parents provided demographic information and ratings of their child’s knowledge of the experimental items on a scale of 1 (child does not understand the word) to 4 (child definitely understands the word). To ensure that we did not measure children’s responses to unknown words, we removed any items where parents marked less than a ‘2’ for the target item. Parents also completed a MBCDI:WS. MBCDI percentile scores ranged from the 1^st^-94^th^ percentile, and children produced between 10–374 words (Fig 2; M_%tile_ = 44.0; SD_%tile_ = 24.2; M_words_ = 92.1; Median_words_ = 61; SD_words_ = 76.3). Children were further grouped into vocabulary groups according to median split of productive vocabulary for subsequent analyses.

**Cognitive Assessment.** After the completion of the experimental task, all children completed the Cognitive subtest of the Bayley Scales of Infant and Toddler Development (Bayley-III) [38]. Because we were interested in including children who were exhibiting normal cognitive development, we excluded any child who fell more than 1.5 SD below the mean standard score (<85). This criterion led to the exclusion of one child. Remaining standard scores ranged between 85–145 (M = 112.2; SD = 13.7)

**Experimental procedure.** The child was seated in a car seat in front of a 17” computer monitor with an eye-tracking camera mounted underneath. Auditory stimuli were presented via a speaker placed behind the monitor. The parent/guardian sat next to the child and one experimenter sat on the other side of the child. Another experimenter remained out of view behind a curtain and controlled the eye-tracking apparatus. Parents were instructed not to name the images on the screen.

The tracker was calibrated with a five-point procedure using a looming bull’s-eye image paired with a whistling sound before proceeding to the experimental task. Each trial began with the presentation of a small, colorful, gaze-contingent image (30 x 30 pixels) presented at the center of the monitor. After the child fixated on this central image, it disappeared and the target and distractor appeared on the left and right sides of the screen for 2000 ms in silence. This silent preview served to familiarize the toddler with the object images and their locations.

Next, before the onset of the target label, a 100 x 100-pixel image (e.g. a yellow happy face, or smiling flower) appeared at the center of the monitor, and a pre-recorded word, “Look!” was played. The purpose of this gaze-contingent central image was to ensure that the trial did not proceed until the child was fully attending to the screen. The entire stimulus array (comprising central, right and left images) fell within 5° visual angle–i.e., within the 5–10° useful visual field typically cited in visual search studies [39]. Therefore, this stimulus arrangement allowed us to probe the child’s visual attention without taxing working memory resources for the placement of each image. Fig 1 illustrates the arrangement of stimuli.

Once the child had fixated the central image for 100 ms, the central stimulus disappeared and target image was named (e.g. *Car*!) followed by a brief positive message (e.g. *Great job*!). Target and distractor images remained on the screen for 4000 ms post label-onset. Each image appeared four times across the experiment–twice as the unlabeled distractor, twice labeled as the target. Additionally, each image appeared twice paired with a semantically related image (one time each as target and distractor), and twice with a semantically unrelated image (once as target and distractor). The location of each image was also counterbalanced, such that it appeared equally on the left and right side of the screen. This arrangement yielded a completely balanced within-subjects design that ensured that each image appeared in related and unrelated experimental conditions, on both sides of the screen, and as a target and distractor with equal likelihood. There were 24 experimental trials, with 12 related trials and 12 unrelated trials, distributed across four blocks of six trials. Between each block, children saw larger images containing popular characters, and they heard enthusiastic statements (e.g., *Those were some cool pictures*, *Let’s see some more*!). The entire procedure lasted approximately 5–10 minutes.

#### Apparatus and recording procedures of eye-movement data

Eye movements were recorded monocularly from image onset to image offset at 500 Hz by an SR-Research Eyelink 1000+ eye-tracker and binned into 50 ms intervals for plotting and analysis. Areas of interest were defined as the two 400 x 400-pixel areas corresponding to the locations of the Target and Distractor images.

### Approach to analysis

#### Measurement of vocabulary size and structure

Toddlers were assigned to either a High (N = 40) or Low (N = 39) productive vocabulary group as determined by a median split of MBCDI productive vocabulary score. We then calculated three metrics of vocabulary structure detailed below.

**Category-level structure (Category Density)**. The procedure for measuring semantic category density followed prior studies[10,11]. The semantic density measurement procedure was as follows: using the child’s MBCDI report, we first calculated the total proportion of words said in each of the six categories from which the experimental items were drawn. Each child’s category proportions were then rank ordered from highest to lowest, and the child’s top three were assigned to a High-Density condition, while the bottom three were assigned to a Low-Density condition. If there were any ties in the #3 and #4 rank, then both categories were assigned to the HIGH condition. Table 1 reports the proportion of participants who were assigned to high and low semantic density across categories.

**Table 1:** Proportion of participants with high or low semantic category density across all six categories.

Category	High	Low
ANIMALS	.47	.53
BODY-PARTS	.58	.42
CLOTHING	.13	.87
DRINK	.54	.46
FRUIT	.67	.33
VEHICLES	.38	.62

**Word-level structure (Degree).** Word- and lexicon-level structure was calculated using graph-theoretic models of each participant’s unique lexico-semantic network following established techniques[16,23]. The nodes in each child’s network were determined according to nouns from the MBCDI that they were reported to produce. Following prior precedent, semantic links between pairs of nodes were created if the associated concrete nouns shared at least two perceptual and/or functional features according to a combined set of feature production norms comprising all nouns in the MBCDI [27].

For the word-level structure metric (degree), we calculated the degree as the number of links shared with all other words in the child’s lexical network for each individual experimental item separately. For example, for the word “dog,” a word degree of zero would indicate that, based on their MBCDI assessment, the child did not say any semantic neighbors that share semantic feature overlap of that individual word, whereas a higher degree, such as 5, would indicate the child produces five other semantically related words.

**Lexicon-level structure (Global Clustering Coefficient).** We measured lexicon-level semantic structure using global clustering coefficient, which is a graph-theoretic metric of the overall semantic connectivity of each child’s lexico-semantic network. Global clustering coefficient calculates average level of connectivity between words across the lexicon, and is calculated as the total number of closed triangles in the network divided by the number of connected triples [40]. A connected triple is defined as any set of three nodes that are connected by semantic links (e.g. cat-dog, cat-mouse), and such a triple is furthermore defined as a closed triangle if all three nodes are directly connected to each other, resulting in a triangle shape (e.g. car-truck, car-helicopter, truck-helicopter). Global clustering coefficient values range from 0 to 1, with 0 indicating that the child’s lexicon has no connected triples, and 1 indicating that all triplets are closed. Note, that it is possible to have a number of individual words with a high degree, indicating many pairwise connections, yet still have a low global clustering coefficient, indicating that the neighbors of individual words do not connect to each other (yielding no / few closed triangles).

#### Analysis of eye-movement data

Children completed both experimental conditions (semantically related and unrelated trials) during the same session. Based on prior research, we hypothesized that different mechanisms (facilitation vs. interference) should lead to relatively different gaze behaviors in each condition, and with each task including relatively different task demands. Our primary hypotheses surrounded the influence and interaction of semantic structure and size on lexical processing in each condition separately. Therefore, rather than directly compare conditions (and the resultant 3-way interactions with vocabulary size and structure, which would necessitate a significantly larger sample), we carry out statistical analyses on related and unrelated conditions separately. Within each condition, we explore the interaction of vocabulary size (as median split vocabulary group) and structure (Degree, Category Density and GCC).

Timecourse plots are included to illustrate the total proportion of fixations to the target and distractor items over all participants to highlight fine-grained patterns of processing. Because children initially fixated towards a central stimulus to initiate the trial, these proportions in fixation include looks outside of the interest areas in their denominators. This calculation results in time course plots where fixations to the Target and Distractor start at 0, indicating that children were not fixating on either image. Then, we carry out a statistical analysis of gaze preference over a time window from 300 to 2,000 ms post label-onset. The chosen time window is commonly used in eye-tracked lexical recognition tasks in children of this age [30]. Our measure of accuracy is calculated using log-gaze proportion ratio, which is calculated as the log proportion of fixations to the Target divided by proportion of fixations to the Distractor [log (P_Target / P_Distractor)]. Thus, this log-gaze proportion measure represents the relative preference for viewing the target over that of the distractor image–with zero values representing equivalent Target / Distractor looking, log-proportions less than 0 indicating a distractor preference, and log-proportions greater than 0 indicating a target preference. Log-gaze proportion measures have been adopted by a number of adult and infant eye-tracking researchers, as they provide a linear transformation that reduces problems with homogeneity of variance and linear independence compared to raw fixation proportions [11,41,42].

We began with a dataset comprising 1892 trials from 79 toddlers. Next, to validate that we measured responses to words that were known to the child, we removed an additional 90 trials (4.8%) where the parent reported that their child did not comprehend the target word, leaving 1802 remaining trials. Then, from the remaining dataset, we removed any trials where participants viewed the screen for less than 20% of the 300–2,000 ms analysis window, following prior precedent[10,11]. This track loss criterion led to the removal of 78 trials (4.3% of the remaining dataset), leaving 1724 trials in the final dataset used for statistical analysis, with 867 trials in the Semantically Unrelated task, and 857 trials in the Semantic Related task. The dataset and analytic code are available at osf.io/4vdgu.

## Results

### Analysis of word recognition performance in unrelated trials

**Fig 3:**
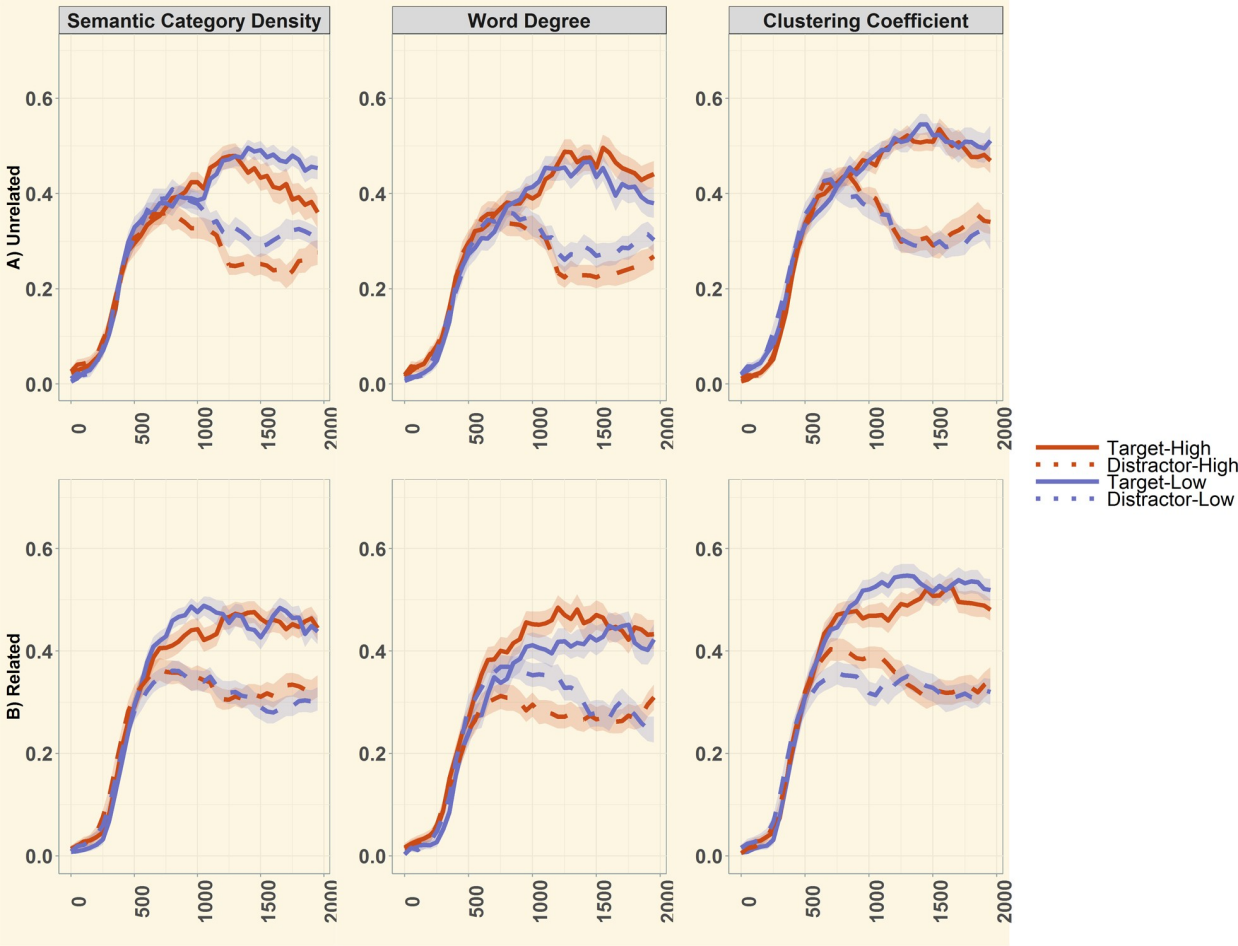
Illustration of fixation proportions to Target and Distractor as a function of semantic structure metrics. Fixation proportions are plotted in 50 ms time bins, and start at the onset of the target label until 2000 ms post label onset, in (A) Semantically Unrelated and (B) Semantically Related conditions. For illustrative purposes, all semantic structure measures are plotted as “high” or “low” values, either according to semantic density assignment, or via median split in the case of continuous measures for word degree and cluster coefficient.

Fig 3 illustrates the timecourse of word recognition as a function of the three semantic structure metrics (semantic category density, word degree and global cluster coefficient) for unrelated and related trials. These plots highlight several important patterns, including that 18-month-olds responded rapidly to the spoken word by directing their looks towards the target approximately 500 ms after spoken word onset. Crucially, the looks towards the target exceeded that of the distractor across all conditions, suggesting that toddlers successfully comprehend the spoken words in this task, and replicating prior findings that 18-month-old children successfully recognize a variety of words in this type of task[43]. There is also apparent variance in the looking patterns across the three semantic structure measures. These patterns are systematically analyzed in the following section.

### Recognition accuracy in the semantically unrelated task

**Fig 4:**
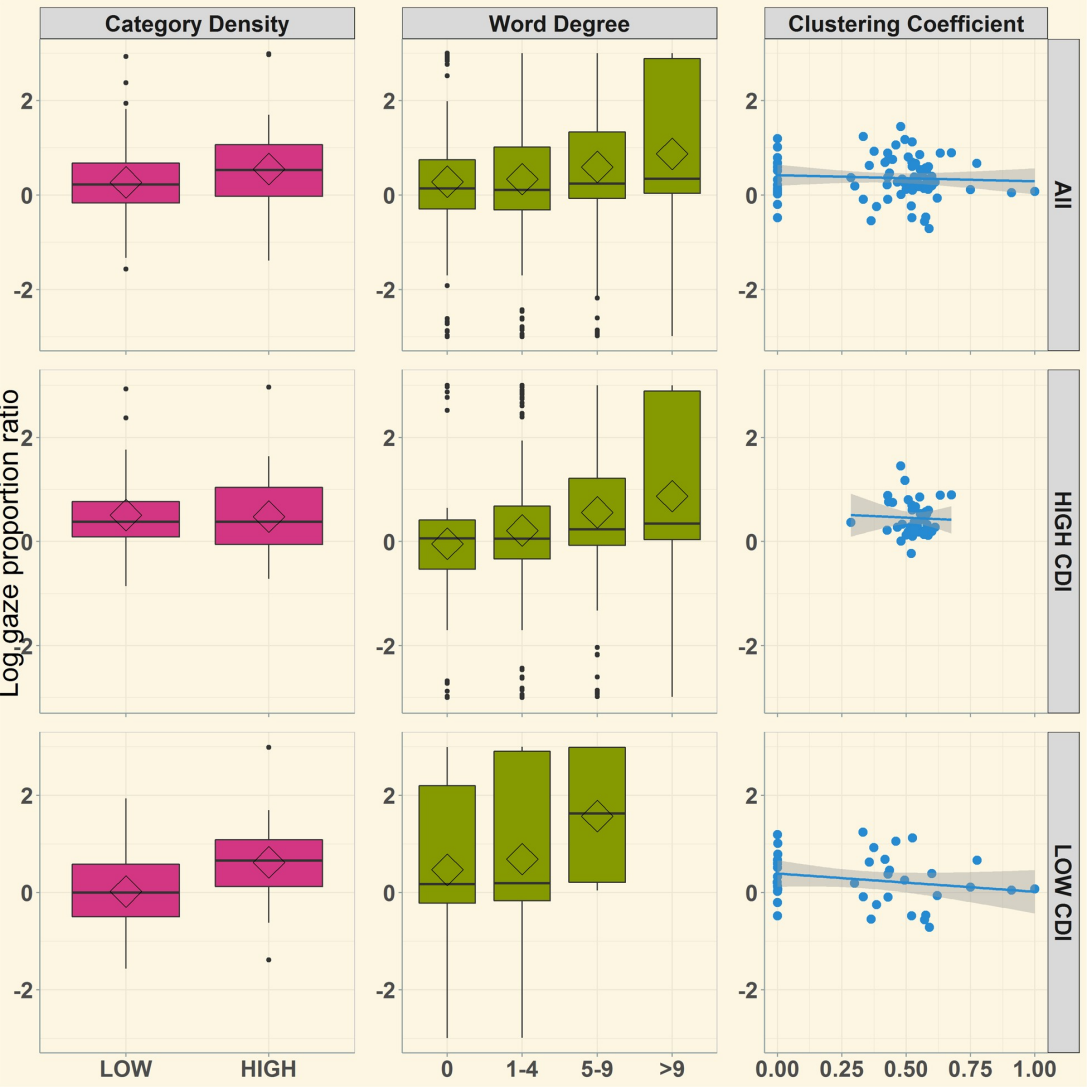
Relation between semantic structure metrics and log-gaze accuracy in the semantically unrelated task. Semantic category density is represented by lower and higher density categories, word degree bars are illustrated in quartile segments of word degree distribution across all target words in the study, and each point on the cluster coefficient graph represents the overall lexical connectivity structure for a single child.

Our goal was to measure the interaction of semantic structure and vocabulary size on word recognition. We calculated performance for each trial as a log-fixation proportion of looks to the Target relative Distractor from 300 to 2,000 ms post spoken word onset (see Fig 4). We then analyzed these log-fixation patterns via linear-mixed effect regression (LMER) using the lme4 library in R. To mitigate potential issues with collinearity and facilitate interpretation of our effects, each fixed effect factor was centered, the categorical variables (category density and vocabulary group) were sum coded (High = -0.5, Low = 0.5), and continuous variables were standardized. We initially sought to test statistical models with participants and items as random effects. However, these models failed to converge, but all models converged when including only items as random effects, therefore we dropped participants as a random effect in all models. The results of the full model are reported in Table 2A and the formula describing the interaction of semantic structure and vocabulary size fixed effects and random effects is listed below:

LogGaze∼(GCC+Degree+CategoryDensity)*Vocab.Group+(1|Items)

**Table 2:** Linear mixed effects regression model for semantically-unrelated trials*.

**A) All participants (N = 79)** **							
	**Estimate**	**Std. Error**		**t-value**		**p-value**	
**(Intercept)**	**0.43**	**0.19**		**2.32**		**.028**	
Vocabulary Group	-.051	0.26		-0.20		.84	
*Semantic category density*	*0*.*21*	*0*.*13*		*1*.*67*		.*096*	
Word Degree	0.17	0.20		0.84		.40	
Global Cluster Coefficient	-0.06	0.13		-0.46		.65	
**Voc. Grp x Sem. Category Density**	**-0.48**	**0.24**		**-2.02**		**0.044**	
Voc. Grp x Word Degree	0.34	0.39		0.87		.39	
Voc. Grp x GCC	-0.067	0.27		-0.24		.81	
Correlation of fixed effects***							
	Intercept		2		3		4
1. Vocabulary Group	.294		.172		.789		.355
2. Semantic category density	-.108		—		-.255		.036
3. Word Degree	.501		-.255		—		-.092
4. Global Cluster Coefficient	-.288		.036		-.092		—
**B) Higher vocabulary group (N = 40)**							
	**Estimate**	**Std. Error**		**t-value**		**p-value**	
**(Intercept)**	**0.43**	**0.17**		**2.56**		**.017**	
Semantic category density	-0.083	0.17		-0.50		.62	
Word Degree	0.081	0.075		1.08		.28	
Global Cluster Coefficient	-0.069	0.25		-0.28		.78	
**C) Lower vocabulary group (N = 39)**							
	**Estimate**	**Std. Error**		**t-value**		**p-value**	
(Intercept)	0.38	0.28		1.36		.18	
**Semantic category density**	**0.45**	**0.19**		**2.40**		**.017**	
Word Degree	0.29	0.42		0.69		.49	
Global Cluster Coefficient	-0.093	0.07		-1.31		.19	

*Panel A illustrates results for all participants, and panels B and C report model outcomes for higher and lower vocabulary groups, respectively. Significant effects are highlighted in bold, marginal effects in italics.

** Exploratory analyses also found that pair-type did not contribute to the model, results can be viewed on osf.io/4vdgu

*** Covariance matrices for high and low vocabulary groups also indicate that semantic structure measures are not correlated, and can be inspected in osf.io/4vdgu

In Table 2A, the significant intercept indicated that 18-month-olds recognized the spoken words by showing a fixation preference for target item. Fixed effect correlations in the model indicated that semantic structure measures were not strongly correlated with each other. The most notable statistical pattern across the entire sample revealed a marginal main effect of category density, which was driven by an interaction with vocabulary size, particularly among children with relatively smaller productive vocabularies.

The significant interaction between vocabulary size and structure corresponds with prior research in this field (reviewed above) that suggested that vocabulary influences word recognition and priming in this age group. Additionally, our covariance effects suggested that some structure measures (degree and global cluster coefficient) were correlated with vocabulary group.

Therefore, to follow up on potentially interesting interactions of vocabulary structure and size, we conducted separate analyses for higher and lower vocabulary participants, (Table 2B and 2C, respectively). This analysis revealed that the category density effect in the full model was driven by the lower vocabulary group. The positive category density estimate for the lower vocabulary group denotes better word accuracy performance for Higher category density items. Thus, the results suggest that increased category density supports word recognition at the onset of word learning.

### Recognition accuracy in semantically related contexts

Fig 3B illustrates the time course of word recognition in the semantically related task as a function of semantic structure at category-, word- and lexicon-level structure. As in the semantically unrelated task, children appeared to understand word meanings across multiple measures of denser and sparser semantic structure, with looks to the target object typically diverging from those to the distractor within 500–1000 ms after word onset. Next, we carried out an LMER analysis using the same approach as in the semantically unrelated task above.

**Fig 5:**
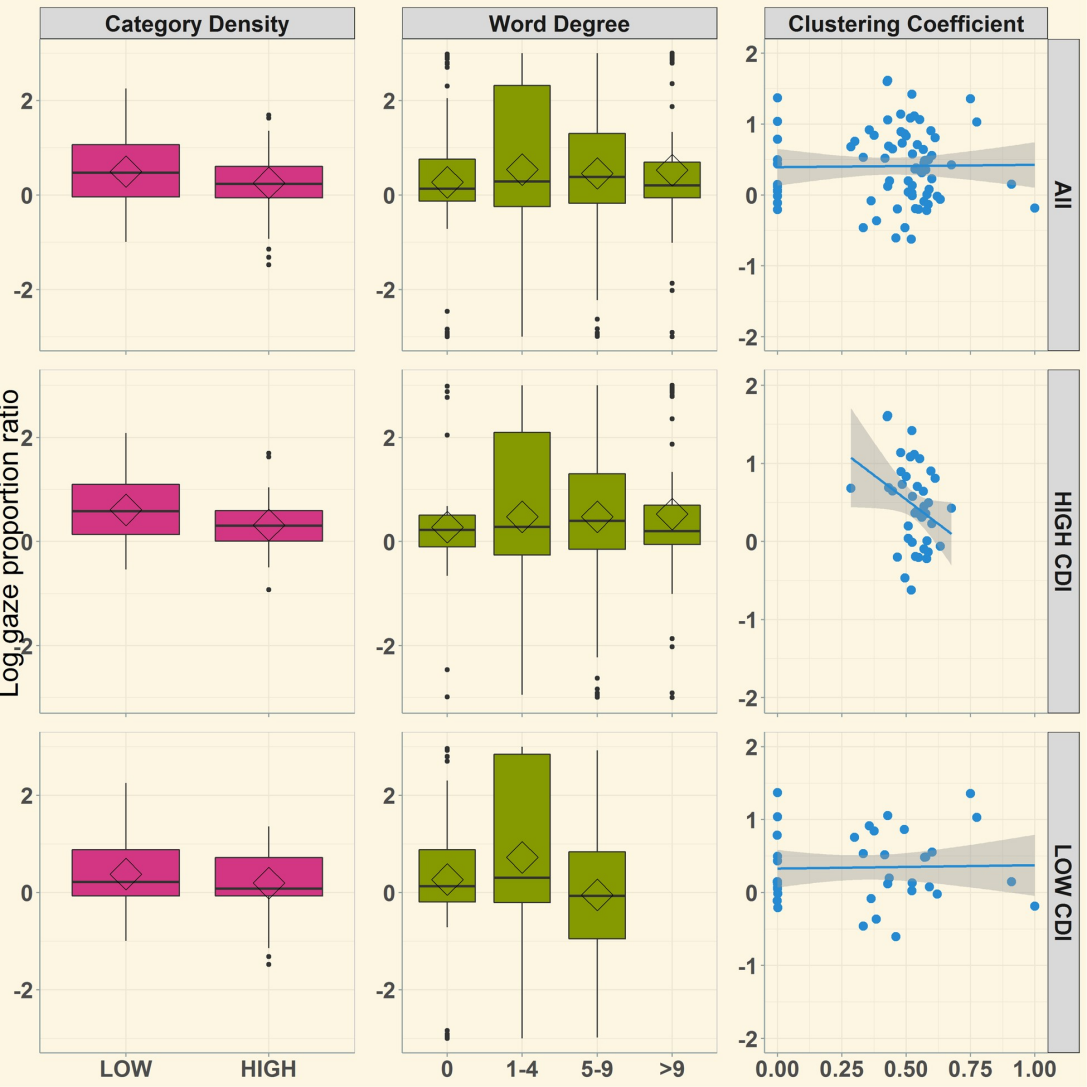
Relations between semantic structure metrics and log-gaze accuracy measure in the semantically related condition. Semantic density is represented by lower and higher density categories, word degree bars represent quartile segments of word degree distribution across all target words in the study, and each point on the cluster coefficient graph represents the overall lexicon structure for a single child.

Fig 5A–5C illustrates log gaze accuracies across all semantic metrics in the semantically related task in overall, higher, and lower vocabulary groups. As before, the full LMER model was specified including random effects of participants and items, and fixed effects that included all measures of productive vocabulary size and structure. Fixed effects were sum-coded, centered, and normalized as in the unrelated task. Model outcomes are outlined in Table 3A. A significant, positive intercept indicated a significant preference to view the target over the distractor. Global cluster coefficient was significantly negatively related to recognition, and category density marginally predicted target preferences. For both measures, higher values (greater lexical interconnectivity and denser categories) predicted greater interference (poorer recognition accuracy). Additionally, vocabulary size marginally interacted with global clustering coefficient. This interaction was explored with separate models for high and low vocabulary groups. Children with higher productive vocabularies also showed successful target recognition, with a significant, positive intercept value (Table 3B). In this group, higher global cluster coefficient values predicted interference–with estimates indicating that greater structure interfered with target recognition. Though children with smaller productive vocabularies successfully recognized the target (as indicated by positive intercept values), no semantic structure measure associated with recognition (Table 3C).

**Table 3:** Linear mixed effects regression model for semantically-related trials*.

A) All participants (N = 79)**					
		**Estimate**	**Std. Error**	**t-value**	**p-value**
**(Intercept)**		**0.59**	**0.14**	**4.11**	**< .0001**
Vocabulary Group		-0.094	0.27	-0.35	.72
*Semantic category density*		*-0*.*23*	*0*.*13*	*-1*.*79*	.*073*
Word Degree		.26	.20	1.26	.21
**Global Cluster Coefficient**		**-0.29**	**0.14**	**-2.02**	**.044**
Voc. Grp x Sem. Category Density		-0.22	0.24	-0.88	.38
Voc. Grp x Word Degree		0.39	0.41	0.94	.35
*Voc*. *Grp x GCC*		*0*.*53*	*0*.*28*	*1*.*87*	.*062*
Correlation of fixed effects***					
		Intercept	2	3	4
1 Vocabulary Group		.405	.169	.791	.362
2. Semantic category density		-.139	—	-.246	-.024
3.Word Degree		.679	-.246	—	-.078
4.Global Cluster Coefficient		-.377	.024	-.078	—
**B) Higher vocabulary group (N = 40)**					
		**Estimate**	Std. Error	**t-value**	**p-value**
**(Intercept)**		**0.64**	**0.14**	**4.56**	**< .0001**
*Semantic category density*		*-0*.*32*	*0*.*16*	*-1*.*96*	.*052*
Word Degree		0.079	0.071	1.11	.27
**Global Cluster Coefficient**		**-0.55**	**0.25**	**-2.22**	**.027**
**C) Lower vocabulary group (N = 39)**					
		**Estimate**	**Std. Error**	**t-value**	**p-value**
**(Intercept)**		**0.56**	**0.25**	**2.27**	**.025**
Semantic category density		-0.12	0.19	-0.64	.52
Word Degree		0.48	0.44	1.10	.27
Global Cluster Coefficient		-.024	0.074	-0.32	.75

*The first panel illustrates LMER results for all participants. Panels B and C convey model results for higher and lower vocabulary groups, respectively. Significant effects are highlighted in bold, marginal effects (p < .1) are in italics.

** Exploratory analyses also found that pair-type did not contribute to the model, results can be viewed on osf.io/4vdgu

*** Covariance matrices for high and low vocabulary groups also indicate that semantic structure measures are not correlated, and can be inspected in osf.io/4vdgu.

## Discussion

The adult lexicon encodes semantic structure that can be described at multiple granularities, each of which can differentially influence lexical processing. How does this structure emerge in development and what effect does it have on lexical activation and processing? One possibility is that this structure has an impact on processing throughout development, irrespective of the learner’s vocabulary size or age. Alternatively, the interplay of semantic structure on language processing may interact with the size of the child’s lexicon. The middle of the second year of life is a particularly interesting period to explore these questions. Children at this age are often at the cusp of a period of rapid vocabulary growth, and have large individual variation in lexical size. Moreover, prior studies had indicated that 18- to 21-month-old children as a group do not demonstrate semantic priming effects, while others had observed priming effects in 18-month-old children with relatively larger productive vocabularies only. Therefore, children of this age have ideal productive vocabulary characteristics to explore the impact of emerging semantic structure on language processing.

We tested children on two interleaved tasks that were designed to tap into different mechanisms of lexical facilitation and interference during word recognition. The first task (semantically unrelated trials) sought to measure how facilitatory mechanisms of lexical activation are impacted by productive vocabulary size and structure. Prior research had suggested that greater category density in 24-month-olds facilitated known word recognition [10]. Our findings replicate and extend these effects to 18-month-olds, by finding that category density similarly supports word recognition in younger children, who understand and produce fewer words than their older peers. Curiously, this effect in 18-month-olds was largely driven by children with relatively smaller productive vocabularies. This interaction with vocabulary size, at face value, might suggest that this facilitatory effect of semantic structure should disappear as children learn more words. However, prior work in 24-month-olds (who have larger vocabularies than 18-month-olds) finds that semantic category density also supports lexical recognition[10]. Future work is needed to explore how semantic structure interacts with age and vocabulary. Accordingly, we are engaged in a longitudinal project to attempt to precisely tease apart influences of maturation and vocabulary size and structure on lexical processing. Another hopeful implication of this vocabulary effect suggests that toddlers with early language delays (such as late-talkers) might be especially responsive to semantically structured vocabulary interventions.

The second task (semantically related trials) sought to measure how categorically related objects interfere with word recognition as a function of vocabulary size and structure. Here, prior research had indicated that 18-month-olds with relatively larger vocabularies were more likely to show priming among word meanings. Our findings shed new insights into these prior findings by suggesting that semantic co-activation between word meanings may be driven by increasing semantic connectivity across the child’s entire productive lexicon. As in prior priming studies, these effects are driven by toddlers with relatively larger productive vocabularies. Thus, while semantic category density supported word recognition in 18-month-olds with smaller productive vocabularies, overall lexical connectivity interfered with word recognition for toddlers with relatively larger vocabularies. One explanation for this pattern is that facilitation was driven by having many nearby semantic neighbors, whereas interference was driven by higher-level structure that incorporates both nearby and distant semantic connections.

The alternating impact of lower- and higher-level structure on facilitation and interference can be tied to notions of “strong” vs. “weak” lexical activation within a word’s neighborhood [1]. Chen and Mirman (2012) posit that lexical facilitation and inhibition vary with the semantic structure of a target word’s neighborhood. They indicate that, *“*if particular patterns of neighbor clustering lead the neighbors to enhance one another’s activation, then they will tend to have more inhibitory (or less facilitative) effects; in contrast, if they do not accentuate one another’s activation, then the cumulative effect on the target will be more facilitative” (p. 426). In other words, this account suggests that interconnected lexicons/neighborhoods (such as those with higher clustering coefficient) interfere with lexical processing. This prediction mirrors the pattern in our semantically related trials, where our lexicon-level measures of structure correlated with greater interference. Similarly, the Chen and Mirman (2012) account also predicts that neighborhoods with “diffuse” or “weak” activation, would show facilitative effects in target recognition. This idea is consistent with the pattern of findings in semantically unrelated trials, where word recognition was facilitated for items in larger local neighborhoods (with higher semantic density) particularly for children with smaller lexico-semantic networks, but not for those with larger vocabularies, or greater lexical connectivity structure.

More generally, the Chen and Mirman (2012) account generates some productive hypotheses regarding how lexical facilitation and interference could shift within individual children as lexicon structure changes across development. First, developmental changes in structure and lexical dynamics are plausible. For example, words with higher degree tend to enter toddler’s lexicons at younger ages[16], and as the lexicon expands, semantic neighborhoods must similarly grow in size, leading to potentially greater opportunities for “strong” clusters of lexico-semantic activation to influence processing. We speculate that potential developmental shifts in neighborhood connectivity may lead to stronger activation patterns, which would correspondingly increase interference effects in processing. This idea is similarly supported by simulations of phonological competition that suggest inhibitory mechanisms in lexical activation emerge as the lexicon grows [44].

This investigation suggests multiple additional avenues for future research. For example, fundamental questions remain regarding how these early processing dynamics are sculpted by maturational and experiential factors, such as recognition of structure in the child’s own environment, or the amount or variety of language input. For example, children’s vocabulary structure can influence attentional biases in learning, such as in cases where knowing many solid objects can support a shape-bias in word learning[45–47]. Findings like these raise interesting questions regarding how the child’s physical environment and their interaction with that environment shapes language learning. Exciting recent technological advances that enable detailed analyses of children’s interactions in their physical environments hold great promise for unlocking discoveries in this area[48,49]. Lexico-semantic processing patterns in children with relatively small lexicons may also share similarities to adults who are starting to learn a new language, or who are encountering a novel set of concepts. Therefore, understanding the developmental trajectories of how these factors interact as the lexicon grows in the early years will be crucial for building rich models of language growth and representation across the lifespan.

These questions also raise several limitations in the current research. First, we must consider that one reason for the difference in patterns between high and low productive vocabulary groups is the possibility that our measures may not have the sensitivity to detect subtler effects in children with smaller vocabularies. However, recent discoveries suggest that under certain cases it is possible to measure semantic priming effects via eye-tracking in infants as young as six to nine months[36]. A full picture of whether and how semantic structure may interact with processing in infants and toddlers who are only beginning to learn word meanings will ultimately require data from multiple converging methods. Even within the same general method (gaze paradigms), numerous design choices may potentially change the pattern of effects[50]. For example, in an interest of balancing general toddler interest across image pairs, we chose to use a yoked-pair design, rather than a random selection of image pairs. Further research is necessary to explore how design choices impact findings across studies.

In sum, our findings suggest that early semantic structure is only starting to influence lexical processing for 18- to 20-month-old children who already have a sizeable lexicon. By combining metrics of semantic detail in the lexicon with measures of language processing, this investigation highlights several novel insights into mechanisms of early facilitation and interference at multiple levels of granularity in the lexicon. These findings motivate a need to use longitudinal methods to develop a full accounting of the dynamic interplay of semantic structure and language processing skills across development. While we are currently collecting such a dataset, in the meantime, our current data provide an early glimpse into the emergence of interactions between semantic structure and lexical recognition skills.
